# Supplementary material for: Combining Novel Biomarkers for Risk Stratification of Two-Year Cardiovascular Mortality in Patients with ST-Elevation Myocardial Infarction
Source: J Clin Med. 2020 Feb 18;9(2):550. doi: 10.3390/jcm9020550 (PMC7073894; doi:10.3390/jcm9020550)
Supplement: Supplementary file 1 [file jcm-09-00550-s001.pdf]

Supplement Table 1. Gehan's Wilcoxon and log-rank analysis of risk factors for CVD in patients with STEMI during the follow-up period

Variable	Z_V-statistics (basing on Gehan's Wilcoxon test)	*LR-statistics* (basing on log-rank criteria)
LVEF < 60%	Z_V=2.308; (p=0.021)	LR=4.211; (p=0.040)
NT-proBNP> 2141 pg/ml	Z_V =2.748; (p=0.006)	LR=8.807; (p=0.003)
ST2 > 27.2 ng/ml	Z_V =2.967; (p=0.003)	LR=9.549; (p=0.002)
Ptx-3 > 169 ng/ml	Z_V =2.807; (p=0.005)	LR=8.284; (p=0.004)
Age > 65 years	Z_V =2.311; (p=0.021)	LR=4.451; (p=0.035)
Gender	Z_V =1.784; (p=0.074)	LR=3.781; (p=0.052)
Troponin I > 1750 ng/ml	Z_V =1.951; (p=0.051)	LR=3.812; (p=0.051)
